# Validity and reliability study of the Turkish adaptation of the Food Craving Acceptance and Action Questionnaire

**DOI:** 10.34172/hpp.42811

**Published:** 2024-10-31

**Authors:** Kerim Kaan Gokustun, Nurcan Yabanci Ayhan

**Affiliations:** ^1^Malatya Turgut Ozal University, Department of Nutrition and Dietetics, Faculty of Health Sciences, Malatya, Turkey; ^2^Ankara University, Department of Nutrition and Dietetics, Faculty of Health Sciences, Ankara, Turkey

**Keywords:** Acceptance, Food addiction, Food cravings, Eating disorders, Willingness

## Abstract

**Background:**

The aim of this study is to evaluate the validity and reliability of the Food Craving Acceptance and Action Questionnaire (FAAQ) in university students.

**Methods:**

The study included 394 undergraduate students at Ankara Yıldırım Beyazıt University. The study included individuals who volunteered to participate, were at least 18 years old, and did not have any severe psychological issues. Explanatory and confirmatory factor analyses of the scale were conducted by dividing the data set into two groups. Cronbach’s α coefficient was analyzed and a test-retest was conducted with 94 students.

**Results:**

It was determined that the Food Craving Acceptance and Action Questionnaire had 2 factors (acceptance and willingness). The fit values of the scale were found to be CMIN/df=2.26; GFI=0.92; AGFI=0.87; CFI=0.85; RMSEA=0.08. The acceptance, willingness subscales and total Cronbach α coefficients of the scale were 0.761, 0.716 and 0.761, respectively. Intraclass correlation coefficient (ICC) values were 0.84, 0.81 and 0.80 for acceptance, willingness and total scale score, respectively. Statistically significant negative correlations were found between the ‘acceptance’, ‘willingness’ subscale and total DEBQ, FCQ-T scores, subscale scores and mYFAS 2.0 symptom count (*P*<0.05).

**Conclusion:**

The FAAQ was found to have a two-factor structure and the fit values were found to be within the acceptable range. The age range for university students is considered to be that of adults, and this scale can also be applied to adults in general.

## Introduction

 Food cravings are defined as the intense urge to consume any food or meal.^1,2^ Food cravings are a common phenomenon in adults.^3^ It was reported that almost all women and 68.0% of men had food cravings.^4^ Food cravings are usually unintentional. Individuals tend to consume more food to avoid negative feelings and thoughts.^5^ Especially in situations where individuals are fixated on the past, unable to live in the present, and emotionally fragile, individuals are more sensitive to thoughts and preoccupations associated with food consumption and food intake rises.^6^ Food cravings can lead to emotional eating behavior, eating behavior disorders such as binge eating behavior, and many health problems such as obesity.^5,7^ Lifestyle changes are very important for the prevention and treatment of these problems caused by food cravings. However, it is very difficult to attain behavioral change in individuals. For this reason, some psychological models and theories are utilized to facilitate lifestyle changes. One of these models and theories is Cognitive Behavioral Therapy.^8^

 Cognitive Behavioral Therapy is a treatment that evaluates the manner in which our thoughts affect our emotions and behaviors. This therapy explains human behavior by focusing on social, emotional, developmental, cognitive and behavioral theories.^8^ Acceptance and Commitment Therapy (ACT) is an important model within the Cognitive Behavioral Therapy theory. It includes the basics of acceptance and mindfulness.^9^ ACT aims to direct the behaviors of individuals by struggling with these feelings and thoughts without changing the negative feelings and thoughts in individuals.^10^ In a study, it was reported that individuals with binge food craving episodes can control their urge, increase their physical activity levels and achieve sustainable body weight loss when they accept these episodes without suppressing or changing them and direct their behaviors accordingly.^11^

 Acceptance of food-related thoughts and the willingness to improve eating behaviors beyond the framework of these thoughts can help to develop effective methods for the treatment of obesity. The Food Craving Acceptance and Action Questionnaire (FAAQ) was developed to determine the extent of acceptance of disturbing feelings, thoughts and experiences about food and willingness to practice healthy eating behaviors despite these feelings, thoughts and experiences. The aim of this study is to conduct the Turkish adaptation of the FAAQ in university students.

## Materials and Methods

###  Participants

 The study was conducted on a total of 394 volunteer university students, 102 (25.9%) males and 292 (74.1%) females, who received undergraduate education at different faculties of Ankara Yıldırım Beyazıt University (Health Sciences, Engineering and Natural Sciences, Law, Dentistry) between February and November 2020. In validity and reliability studies, since the sample size is recommended to be at least 10 times the number of items, the study was conducted with 394 university students.^12^ The study included individuals who volunteered to participate, were at least 18 years old, and did not have any severe psychological issues, while pregnant and breastfeeding students, individuals who incompletely filled out the questionnaire or who were thought not to have completed the questionnaire reliably were excluded from the study.

###  Data collection tool

 While the data of the study was collected face-to-face from some students due to the pandemic, it was obtained from some students using an online platform (Google Surveys) that could be easily administered as of April 2020. The link to the questionnaire form was delivered to the individuals via e-mail or social media (Instagram, Facebook and WhatsApp) applications. The questionnaire form consists of 5 sections, including descriptive data of the individuals, the FAAQ, the Food Craving Scale (FCQ-T), the Dutch Eating Behavior Questionnaire (DEBQ) and the Modified Yale Food Addiction Scale Version 2.0 (mYFAS 2.0).

###  Descriptive characteristics of individuals 

 Descriptive variables such as age, gender, faculty of study and class of study were included.

###  Food Craving Acceptance and Action Questionnaire 

 The FAAQ was developed by Juarascio et al.^11^ The FAAQ consists of 10 items and two subscales. In the ‘acceptance’ subscale, there are 4 items (questions 4, 6, 7 and 9) that assess the acceptance of feelings, thoughts and cravings related to food without suppressing or trying to change them. The ‘willingness’ subscale consists of 6 items (questions 1, 2, 3, 5, 8, 10) that assess the individual’s willingness to direct themselves towards a healthy diet despite the feelings, thoughts and excessive desire associated with food. The scale was designed as a 6-point Likert scale. The items were evaluated between 1 and 6 points (1 = very rarely true, 6 = always true) and questions 4, 6, 7 and 9 were inversely scored. Higher scores reflect a higher acceptance of the motivation for adequate and balanced nutrition. The Cronbach α of the scale was found to be 0.66.^11^

###  Food Craving Scale 

 The Food Craving Questionnaire (FCQ-T) was developed by Cepeda-Benito et al^13^ to evaluate food craving in individuals. This scale consists of 39 items and 9 subscales. The FCQ-T was developed as a 6-point Likert scale. The scale is assessed between 1 and 6 points (1 = Never, 6 = Always). High scores on this scale indicate the development of food cravings.^13^ The Turkish translation of the FCQ-T was conducted by Muftuoglu et al.^2^ The Cronbach α coefficient of the scale was found to be 0.97.^2^

###  Dutch Eating Behavior Questionnaire 

 The DEBQ was developed based on emotional eating, external eating and restrictive eating behaviors.^14^ The DEBQ has 33 items and is prepared as a 5-point Likert scale and is scored between 1 and 5 points (1 = never, 5 = very often).^14^ The validity and reliability study of this study was conducted by Bozan et al (Cronbach α = 0.94).^15^

###  Modified Yale Food Addiction Scale Version 2.0 

 The Yale Food Addiction Scale Version 2.0 was developed by Gearhardt et al based on the substance use disorders in DSM-V.^16^ The scale includes 35 questions assessing 11 criteria for substance use disorders. The mYFAS 2.0 is a short version of the Yale Food Addiction Version 2.0 and includes 13 items that measure the criteria. The scale was prepared as an 8-point Likert scale (0 = never, 7 = every day). The scale is evaluated between 0-11 points.^17^ The validity and reliability of this scale in adults was conducted by Tok and Ekerbiçer (Cronbach α = 0.72).^18^

###  Turkish Adaptation Protocol

####  Translation-Back Translation

 For the validity and reliability of our study, a translation-retranslation study was initially conducted. The scale was translated into Turkish by 3 professionals who are fluent in English and Turkish. After it was translated into Turkish, a pilot study was conducted with 30 students in order to make the incomprehensible or unclear items of the scale more understandable and clear. Necessary adjustments were made in accordance with the feedback from the students and the scale was made more intelligible. The final version of the scale was translated back into English and the Turkish and English forms of the scale were compared by 3 experts and evaluated in terms of meaning and grammar.^19^

###  Validity, reliability and statistical analyses of the FAAQ

####  Dimensionality and confirmatory factor analysis

 Exploratory factor analyses were conducted to validate the FAAQ. Kaiser-Mayer-Olkin (KMO) and Bartlett’s sphericity test were performed to evaluate sample adequacy for factor analysis. A KMO value of 0.7-0.8 means that the sample size is ‘moderate’; 0.8-0.9 indicates good; > 0.9 indicates very good.^20^

 After the sampling adequacy of the scale was tested, the data set was divided into two parts for factor analysis. Exploratory factor analysis was conducted for the individuals in the second part (with 198-394 IDs) and confirmatory factor analysis was conducted for the individuals in the first part (with 1-197 IDs). The exploratory factor analysis was conducted via SPSS 23 software (IBM Inc., Chicago, IL, USA) statistical package program. Varimax rotation was performed to determine the loadings of the factors in the exploratory factor analysis. Eigenvalues greater than one were considered as factors.

 Confirmatory factor analysis was conducted via AMOS 23 software (IBM Corp., Armonk, NY, USA) package program. Standardized estimate coefficient and second order confirmatory factor analysis were reported. For confirmatory factor analysis, fit indices such as CMIN/df, comparative fit index (CFI), root mean square error of estimation (RMSEA), adjusted goodness of fit index (AGFI), goodness of fit index (GFI) were used. CMIN/df < 2.5; GFI > 0.90; AGFI < 0.85; CFI > 0.90; RMSEA < 0.10 values were considered as ‘acceptable fit’.^21,22^

###  Reliability

 Cronbach-α coefficient of the scale and subscales was analyzed for the reliability of the scale. A Cronbach-α coefficient of 0.60-0.79 is considered to be ‘highly reliable’, while a coefficient of 0.80 and above is considered as ‘high reliability’.^20^

###  Test-retest

 In order to evaluate the reliability of the scale, the scale was re-administered to 94 participants within 14-28 days after completing the first scale. Intraclass correlation coefficient (ICC) analysis was performed to evaluate the difference between the two practices. An ICC value of 0.80 and above means that the reliability of the scale is ‘good’.^23^

###  Construct validity

 In order to determine the construct validity of the FAAQ, we examined correlations of theoretically-similar scales (DEBQ, FCQ-T and mYFAS 2.0) with the FAAQ. Correlation analysis was performed between the total scale and subscale scores. Correlations between the scale and subscales were analyzed by Pearson Correlation Analysis.

## Results

Table 1 shows the demographic data of the individuals who participated in the study. 48.5% of the participants were between the ages of 18-20 years; 42.4% were between the ages of 21-23 years. The mean age of males was 21.84 ± 2.55 years; the mean age of females was 20.52 ± 1.96 years. 62.4% of the participants were studying at the Faculty of Health Sciences and 28.2% at the Faculty of Engineering and Natural Sciences. In our study, 28.4% of the participants were studying in the 2^nd^ grade.

**Table 1:** The baseline characteristics of study subjects (n = 394)

**Variable**	**Total**
Age (year), No. (%)	
18-20	191 (48.5)
21-23	167 (42.4)
24-26	28 (7.1)
27 and more	8 (2.0)
(Mean ± SD) (Min-Max)	20.86 (2.20) (17-34)
Faculty	
Health Sciences	246 (62.4)
Engineering and Natural Sciences	111 (28.2)
Law	19 (4.8)
Dentistry	18 (4.6)
Class	
1^st^ Grade	99 (25.1)
2^nd^ Grade	112 (28.4)
3^rd^ Grade	80 (20.3)
4^th^ Grade	103 (26.2)

###  Dimensionality and confirmatory factor analysis

 The KMO value of the scale was 0.72 (chi-square = 590.31; p < 0.01). As the dataset was divided into 2 parts, it was determined that the level of sampling adequacy was ‘moderate’. Since the FAAQ had two factors with eigenvalues greater than 1, it was determined that this scale had two factors. The number of items collected under factor 1 was 4 (items 4, 6, 7, 9); the number of items collected under factor 2 was 6 (items 1, 2, 3, 5, 8, 10). Factor 1 was defined as ‘acceptance’ and factor 2 as ‘willingness’. Factor loadings ranged between 0.54 and 0.86. The eigenvalue of the ‘acceptance’ subscale was found to be 3.35 and the variance percentage was 33.49%. The eigenvalue of the ‘Willingness’ subscale was found to be 1.90 and the variance percentage was found to be 18.96%. These two factors explain 52.45% of the total variance (Table 2).

**Table 2:** Evaluation of psychometric properties of the Food Craving Acceptance and Action Questionnaire (FAAQ)

**Items**	**Acceptance**	**Willingness**
FAAQ6	0.861	
FAAQ4	0.764	
FAAQ9	0.738	
FAAQ7	0.667	
FAAQ8		0.693
FAAQ10		0.683
FAAQ1		0.647
FAAQ2		0.635
FAAQ3		0.613
FAAQ5		0.536
Eigenvalue	3.35	1.90
Total variance explained	33.49	18.96
Cumulative variance explained	33.49	52.45

**Figure 1:**
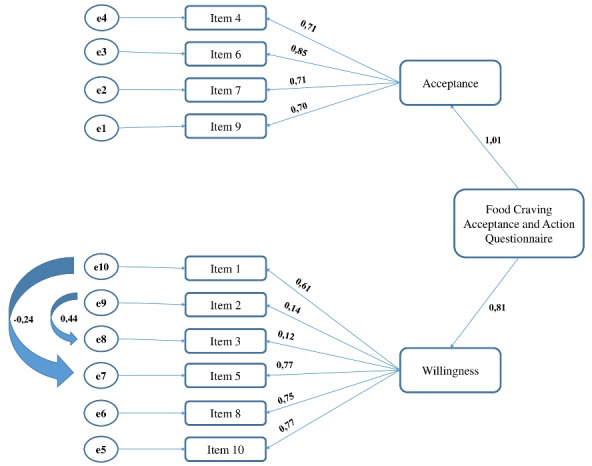
Evaluation of the 2-factor structure of the Food Craving Acceptance and Action Questionnaire (FAAQ) with path diagram

 The evaluation of the 2-factor structure of the FAAQ scale with the path diagram is given in Figure 1. Modifications were made between items 8-9 and 7-10 of this scale. Distribution independent estimation method was carried out. The fit values were CMIN/df = 2.26; GFI = 0.92; AGFI = 0.87; CFI = 0.85; RMSEA = 0.08. In general, the fit indices were ‘acceptable’.

###  Reliability and test-retest

 Distribution of internal consistency values and test-retest results for FAAQ are given in Table 3. The total Cronbach-α coefficient was found to be 0.761, 0.716 for the willingness subscale and 0.761 for the acceptance subscale. In the light of these values, the Cronbach’s α values of the willingness and acceptance subscales and the total scale are ‘highly reliable’. In addition, when the test-retest application was examined, ICC values were 0.84, 0.81 and 0.80 for acceptance, willingness and total scale score, respectively.

**Table 3:** Distribution of internal consistency values and test-retest results for FAAQ

**FAAQ**	**Number of Items**	**Items**	**Cronbach’s-α**	**ICC**
Acceptance	4	4, 6, 7, 9	0,761	0,84
Willingness	6	1, 2, 3, 5, 8, 10	0,716	0,81
Total	10	1-10	0,761	0,80

###  Construct validity

 The correlations between the subscales of the FAAQ and the FAAQ total score and the subscales, total scores and mYFAS 2.0 symptom count of the DEBQ and FCQ-T scales are given in Table 4. Statistically significant negative correlations were found between the ‘willingness’ subscale and total DEBQ, FCQ-T scores, subscale scores and mYFAS 2.0 symptom count (*P* < 0.05). Statistically significant negative correlations were found between the ‘acceptance’ subscale and the restrained eating, positive reinforcements and guilt subscales (*P* < 0.05). Statistically significant negative correlations were found between the total FAAQ score and all subscale and total scale scores except the restricted eating subscale (*P* < 0.05).

**Table 4:** Correlations between the subscales of the Food Craving Acceptance and Action Questionnaire (FAAQ) and FAAQ total score and the subscales and total scores of the DEBQ and FCQ-T scales and mYFAS 2.0 symptom count

**Scales and Subscales**	**FAAQ**					
	**Acceptance**		**Willingness**		**Total**	
	***r***	***P*** ^*^	***r***	***P*** ^*^	***r***	***P*** ^*^
DEBQ						
Restrained eating	-0.182	< 0.001	0.147	0.003	-0.003	0.951
Emotional eating	0.002	0.974	-0.237	< 0.001	-0.219	< 0.001
External eating	-0.025	0.625	-0.214	< 0.001	-0.218	< 0.001
FCQ-T						
Intentions	0.022	0.663	-0.219	< 0.001	-0.187	< 0.001
Positive reinforcements	-0.103	0.042	-0.138	0.006	-0.207	< 0.001
Negative reinforcements	-0.032	0.532	-0.100	0.048	-0.117	0.020
Inadequate control	-0.029	0.566	-0.333	< 0.001	-0.332	< 0.001
Thoughts	0.062	0.219	-0.210	< 0.001	-0.148	0.003
Hunger	-0.045	0.371	-0.156	0.002	-0.179	< 0.001
Emotions	-0.039	0.435	-0.222	< 0.001	-0.237	< 0.001
Stimulants	-0.020	0.692	-0.288	< 0.001	-0.283	< 0.001
Feeling guilty	-0.170	0.001	-0.163	0.001	-0.283	< 0.001
Total	-0.043	0.398	-0.259	< 0.001	-0.274	< 0.001
mYFAS 2.0 symptoms count	-0.015	0.762	-0.351	< 0.001	-0.338	< 0.001

^*^Pearson correlations analysis.

## Discussion

 The results of the exploratory factor analysis of the FAAQ revealed that the scale had two subscales and item factor loadings ranged between 0.54 and 0.86. In the study conducted by Juarascio et al^11^ similar to that study, it was determined that the scale had two subscales and item factor loadings ranged between 0.59 and 0.87. In addition, the factors found in this study were defined as ‘acceptance’ (items 4, 6, 7, 9) and ‘willingness’ (items 1, 2, 3, 5, 8, 10) similar to the study conducted by Juarascio et al.^11^ However, since the confirmatory factor analysis fit index values or the internal consistency coefficient were not sufficient in the Spanish version of this scale, the scale was revised. It was found that the revised version of the scale had a 2-factor structure and item factor loadings ranged between 0.68 and 0.82.^24^ In addition, in a study conducted by Burton Murray et al,^25^ with individuals seeking body weight loss, it was determined that the fit index values of the original version of the scale were not sufficient. Since items 1, 3 and 6 of the scale were not clearly understood by the participants, these items were removed from the scale. Evaluating the 7-item version of the scale, it was found that it showed a 2-factor structure with item factor loadings ranging between 0.42 and 0.86.^25^ In this study, it was found that items 1, 3 and 6 of the scale were clearly understood by the participants; the confirmatory factor analysis fit index values of the scale were in the ‘acceptable’ range; and the CMIN/df value showed a good fit.

 The Cronbach’s alpha coefficient of the scale was determined to be ‘highly reliable’ in all subscales and total scales for university students. In the study conducted by Juarascio et al^11^ with adults, the Cronbach-α coefficient was found to be ‘highly reliable’ for the ‘acceptance’ subscale and the total scale (Cronbach-α coefficient: 0.60 and 0.66, respectively) and ‘high reliability’ for the ‘willingness’ subscale (Cronbach-α coefficient: 0.82). In other validity and reliability studies, the Cronbach alpha coefficient of this scale was found to be similar to our study^24,25^ and higher than the values in the study conducted by Juarascio et al.^11^

 In our study, statistically significant negative correlations were found between the willingness subscale and total FAAQ scores and emotional and external eating scores. These findings indicate that individuals who consume food to suppress negative emotional states such as feeling of sadness, anxiety and loneliness or who consume food depending on external stimuli such as sight and smell do not have adequate healthy eating attitudes and behaviors, and their desire to direct themselves to healthy eating habits is insufficient. In the study conducted by Juarascio et al,^11^ statistically significant positive correlations were reported between the ‘willingness’ subscale and emotional eating and external eating subscale scores. The reason for this discrepancy may be that both university and general population were included in the study conducted by Juarascio et al^11^ and the study was conducted with individuals of different ethnic origins. In addition, in the current study, statistically significant negative correlations were found between the willingness subscale and total FAAQ scores and total FCQ-T, FCQ-T subscale scores and mYFAS 2.0 symptom count. In the study conducted by Burton Murray et al^25^ negative correlations were found between FAAQ subscale and total scores and total FCQ-T similar to our study. In addition, correlations between the Three-Factor Eating Scale and the FAAQ were evaluated in studies; statistically significant negative correlations were found between restrictive eating, emotional eating and uncontrolled eating subscales and total FAAQ, acceptance and willingness subscales.^24,25^ University students are susceptible to external stimuli related to food and are individuals who are likely to consume and prefer food depending on psychological factors.^26,27^ Individuals who have a high willingness to eat healthily can accept their thoughts and cravings about food without changing or suppressing them in any way, and food addiction symptoms may be less likely to be seen in these individuals.

## Limitations

 There are some limitations in our study. First, since the study is based on responses by participants, a certain level of bias may be involved. Secondly, due to the pandemic, the research data were collected both online and through face-to-face interviews. It is considered that the results may be affected by these conditions. Thirdly, the cross-sectional nature of the study does not clearly reveal the direction of the relationships detected in the study.

## Conclusion and Recommendations

 It was found that the FAAQ showed a two-factor structure, the fit index values were at an ‘acceptable’ level and the reliability analyses were ‘highly reliable’. In addition, statistically significant correlations were observed between FAAQ and FCQ-T, mYFAS 2.0 and DEBQ. This scale may be a valid and reliable scale for university students. The age range for university students is considered to be that of adults, and this scale can also be applied. Moreover, the FAAQ can be instrumental in determining body weight loss strategies. It may therefore be beneficial to use the scale for adults targeting body weight loss.

 It should be taken into consideration that eating attitudes and behaviors of university students as compared to adult individuals may be different. Therefore, it would be beneficial to take into consideration the difference between adults and university students in the acquisition of healthy eating habits. Since improving eating behaviors also relies on psychological factors, dieticians and psychologists should collaborate to promote public health and healthy eating habits.

## Acknowledgements

 The study was presented at the X. Nutrition and Dietetics E-congress as a poster presentation.

## Competing Interests

 The authors declare that there are no significant competing financial, professional, or personal interests that might have affected the performance.

## Ethical Approval

 This study obtained protocol and procedure approval from Ankara Yıldırım Beyazıt University Social and Human Sciences Ethics Committee (No: 2020-29). Respondents who agreed to participate in this study were asked to sign an informed consent form.
